# Analogs of the Scorpion Venom Peptide Stigmurin: Structural Assessment, Toxicity, and Increased Antimicrobial Activity

**DOI:** 10.3390/toxins10040161

**Published:** 2018-04-18

**Authors:** Adriana M. S. Parente, Alessandra Daniele-Silva, Allanny A. Furtado, Menilla A. Melo, Ariane F. Lacerda, Moacir Queiroz, Cláudia Moreno, Elizabeth Santos, Hugo A. O. Rocha, Euzébio G. Barbosa, Eneas Carvalho, Arnobio A. Silva-Júnior, Marcelo S. Silva, Matheus de F. Fernandes-Pedrosa

**Affiliations:** 1 Laboratório de Tecnologia e Biotecnologia Farmacêutica, Universidade Federal do Rio Grande do Norte, Natal, Rio Grande do Norte 59010-115, Brazil; adrianamsparente@gmail.com (A.M.S.P.); alessandra.daniele@outlook.com (A.D.-S.); allannyfurtado@hotmail.com (A.A.F.); menillamam@outlook.com (M.A.M.); arianeflacerda@gmail.com (A.F.L.); elizabethcgsantos@gmail.com (E.S.); arnobiosilva@gmail.com (A.A.S.-J.); 2 Programa de Pós-Graduação em Ciências Farmacêuticas, Universidade Federal do Rio Grande do Norte, Natal, Rio Grande do Norte 59072-970, Brazil; euzebiogb@gmail.com (E.G.B.); mssilva.ufrn@gmail.com (M.S.S.); 3 Programa de Pós-Graduação em Bioquímica, Universidade Federal do Rio Grande do Norte, Natal, Rio Grande do Norte 59072-970, Brazil; moacirfqn@gmail.com (M.Q.); claudia.mrn1@gmail.com (C.M.); hugo-alexandre@uol.com.br (H.A.O.R.); 4 Instituto Butantan, São Paulo 05503-900, SP, Brazil; eneas.carvalho@butantan.gov.br; 5 Global Health and Tropical Medicine, Institute of Hygiene and Tropical Medicine, Universidade Nova de Lisboa, 1099-085 Lisbon, Portugal

**Keywords:** antimicrobial peptide, scorpion venom, antiproliferative, antiparasitic, structure-activity relationship, Stigmurin, analog peptides

## Abstract

Scorpion venom is a rich source of biologically active components and various peptides with high-potential therapeutic use that have been characterized for their antimicrobial and antiproliferative activities. Stigmurin is a peptide identified from the *Tityus stigmurus* venom gland with high antibacterial and antiproliferative activities and low toxicity. Amino acid substitutions in peptides without a disulfide bridge sequence have been made with the aim of reducing their toxicity and increasing their biological activities. The purpose of this study was to evaluate the structural conformation and structural stability, as well as antimicrobial, antiproliferative, and hemolytic activities of two peptide analogs to Stigmurin, denominated StigA6 and StigA16. In silico analysis revealed the α-helix structure for both analog peptides, which was confirmed by circular dichroism. Data showed that the net charge and hydrophobic moment of the analog peptides were higher than those for Stigmurin, which can explain the increase in antimicrobial activity presented by them. Both analog peptides exhibited activity on cancerous cells similar to the native peptide; however, they were less toxic when tested on the normal cell line. These results reveal a potential biotechnological application of the analog peptides StigA6 and StigA16 as prototypes to new therapeutic agents.

## 1. Introduction

*Tityus stigmurus* (*T. stigmurus*) is the predominant scorpion in the Northeast region of Brazil. It is considered the leading cause of scorpion accidents in this region, mainly in children and elderly patients [1,2]. Its venom is composed of enzymes, peptides, biogenic amines, amino acids, salts, and other high and low molecular mass proteins, which can either act as toxins or that aid to distribute the toxins in the victim [3,4]. Antimicrobial peptides (AMPs) from scorpions are small cationic molecules that are considered to be the first line of defense against microbes; they show a broad spectrum action against bacteria, fungi, protozoa, and viruses, but can also show activity against cancerous cells [5,6,7]. AMPs interact with microbe cellular membranes and, therefore, their structure is a relevant aspect for their antimicrobial activity and selectivity property. The structural conformation, amphipathicity, net charge, and hydrophobic moment of AMPs are important for determining the peptides’ interaction with microbe membranes [8,9]. AMPs interact with the cellular membrane of microbes, leading to pore formation and cellular lysis; membranes of cancerous cells have different phospholipid content from normal eukaryotic cells, as cancerous cells show a negative net charge membrane due to the phosphatidylserine and o-glycosylated mucins [10]. The ability of these molecules to interact with membranes can decrease the possibility of pathogen resistance development [3,11], which is a growing worldwide problem [12].

Stigmurin is an antimicrobial peptide discovered by our research group in the venom gland transcriptome study of *T. stigmurus* [4]. It is a cationic peptide containing 17 amino acid residues (FFSLIPSLVGGLISAFK-NH_2_), with +1 net charge and hydrophobic moment of 0.571 [13,14], which presented antimicrobial activity in vitro and in vivo, as well as antiproliferative properties in normal and cancerous cells, with low hemolytic activity [13,14]. The rational design of molecules has been seen to potentiate their activity and biotechnological use; the increase in α-helix, cationic character, and hydrophobic moment can empower the antimicrobial activity [15,16,17]. Therefore, two peptide analogs to Stigmurin, denominated as StigA6 (FFSLIPKLVKGLISAFK-NH_2_) and StigA16 (FFKLIPKLVKGLISAFK-NH_2_), where serine and glycine were replaced with lysine, were synthesized in order to enhance their antimicrobial and antiproliferative activities.

## 2. Results

### 2.1. In Silico Evaluation

**Figure 1:**
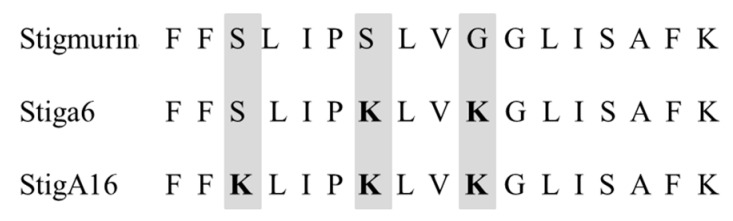
Amino acid sequences for Stigmurin and its analog peptides.

**Figure 2:**
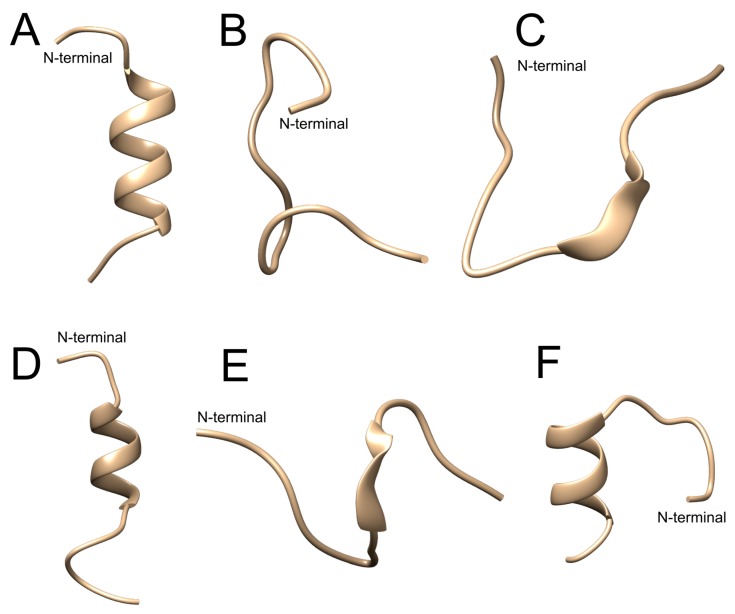
Models for StigA6 and StigA16 obtained by (**A** and **D**, respectively) I-TASSER, Molecular dynamics in water (**B** and **E**, respectively) and in (**C** and **F**, respectively) 50% 2,2,2-trifluoroethanol (TFE).

From Stigmurin, we designed two analog peptides denominated as StigA6 and StigA16 (Figure 1), with higher net charge (+3 and +4, respectively) and hydrophobic moment (0.669 and 0.725, respectively). The models obtained for both peptides using the I-TASSER server showed a helical conformation with some random structure at the N- and C-terminals, as shown in Figure 2A,D for StigA6 and StigA16, respectively.

The obtained models were submitted to molecular dynamics simulation in water medium (Figure 2B for StigA6 and Figure 2E for StigA16). For both analog peptides, the proportion of α-helix conformation shown in the molecular models was reduced along with the random coil conformation proportion increment. When 50% 2.2.2-trifluoroethanol (TFE) was added to the water simulations, the StigA16 model showed an α-helix structure (Figure 2E), while StigA6, at the end of the simulation, showed an attempt to form an α-helix structure (Figure 2C).

### 2.2. Circular Dichroism

**Figure 3:**
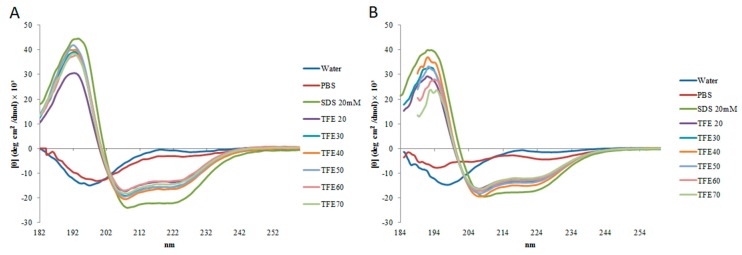
Spectrum of (**A**) StigA6 and (**B**) StigA16 obtained by circular dichroism (CD) analysis in water, sodium phosphate buffer (PBS), sodium dodecyl sulfate (SDS), and TFE (20–70%).

In circular dichroism (CD) analysis, StigA6 and StigA16 showed a similar spectrum. In sodium phosphate buffer (PBS) and water they have a predominantly random structure, but in 20 mM sodium dodecyl sulfate (SDS) and 2,2,2-trifluoroethanol (TFE) they showed a typical α-helix spectrum (Figure 3). These results could also be seen in the deconvolution of the CD spectra (Table 1). StigA6 showed only 4.55% of an α-helix in PBS but showed 66% of an α-helix structure in SDS. StigA16 demonstrated 1.45% of an α-helix structure in water and 58% in SDS.

**Figure 4:**
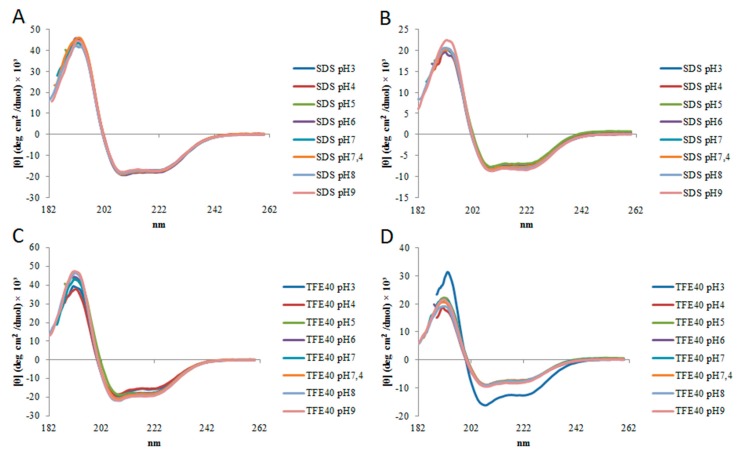
Spectrum obtained by circular dichroism (CD) of (**A**,**C**) StigA6 and (**B**,**D**) StigA16 in 20 mM SDS and 40% TFE under different pH conditions (3–9).

**Figure 5:**
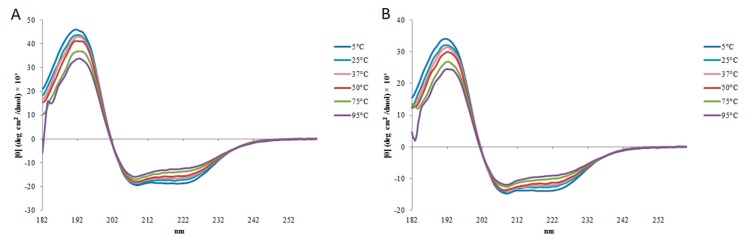
Spectrum obtained by circular dichroism (CD) of (**A**) StigA6 and (**B**) StigA16 in 40% TFE at 5, 25, 37, 50, 75, and 95 °C.

**Figure 6:**
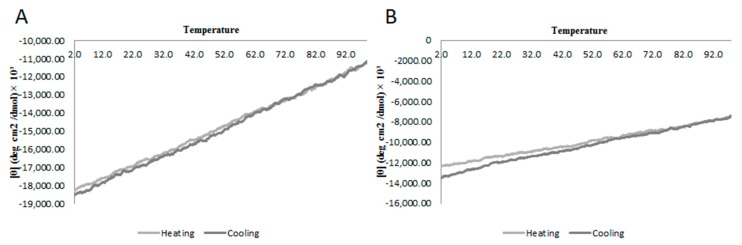
Molecular ellipticity at 222 nm obtained by circular dichroism (CD) of (**A**) StigA6 and (**B**) StigA16, which were heated (grey) from 2 to 98 °C and then cooled back (black).

The secondary structure of StigA6 and StigA16 in SDS and 40% TFE obtained by deconvolution of CD spectrum showed that they maintained the α-helix structure through pH change (3–9) as shown in Figure 4, with the exception of StigA16 at 40% TFE, pH 3, in which it showed a higher helical structure (82%). Concerning the thermal stability, both analog peptides in 40% TFE at 5–98 °C showed a decrease in the ellipticity as the temperature increased, suggesting the occurrence of a temperature-dependent structure loss (Figure 5). Thus, we performed a heating to 92 °C, followed by cooling to 2 °C assay with both analog peptides, in which we could observe that after heating and cooling, both peptides were able to return to a secondary structural conformation pattern very similar to those respectively observed at the beginning of the experiment (Figure 6).

### 2.3. Antimicrobial Activity

StigA6 and StigA16 showed high antimicrobial activity for all Gram-positive and -negative bacteria and yeasts strains tested (Table 2). StigA6 showed minimum inhibitory activity (MIC) between 1.17 and 37.5 µM, while StigA16 showed MIC between 1.17 and 9.38 µM. Interestingly, both analog peptides showed MIC of 1.17 µM when tested against *Enterococcus faecalis* (ATCC 29212). For all bacteria and yeast strains tested, Stigmurin presented higher MIC values than the analog peptides.

### 2.4. Antiparasitic Activity

**Figure 7:**
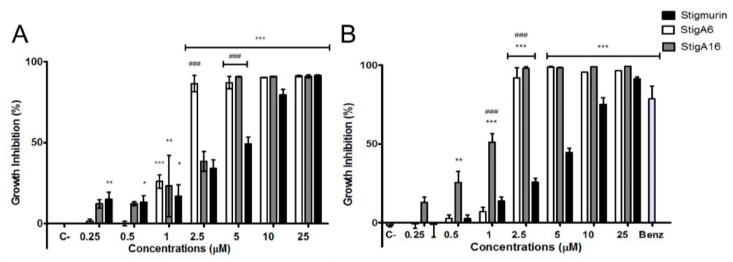
Antiparasitic activity of Stigmurin and its analogs on epimastigote forms of *T. cruzi* after 12 h (**A**) and 24 h (**B**) of incubation. Negative control is represented by C-. Positive control (Benznidazole) is represented by Benz. Values represent mean ± SD (N = 3). *** *p* ≤ 0.0001, ** *p* ≤ 0.001 and * *p* ≤ 0.01 compared to the negative control. ### *p* ≤ 0.0005 compared to Stigmurin at the same concentration. Statistical analysis was performed using ANOVA followed by Tukey test.

After 12 and 24 h of incubation, Stigmurin and its analog peptides showed high antiparasitic activity against epimastigote forms of *Trypanosoma cruzi* (Figure 7A). StigA6 and StigA16 were efficient to inhibit 100% of the parasite growth at a concentration of 2.5 µM after 12 h of incubation, while Stigmurin, at a concentration of 25 µM (which represents a tenfold increase), inhibited 90% of the parasites, indicating that the analog peptides were more efficient than the native peptide. After 24 h incubation at a concentration of 2.5 µM, both analog peptides inhibited 100% of the parasites growth (Figure 7B). However, Stigmurin, at the same concentration, showed no significant epimastigote growth inhibition. Both analog peptides showed higher growth inhibition when compared to Benznidazole. No statistical difference between 12 and 24 h peptide inhibition was found.

**Figure 8:**
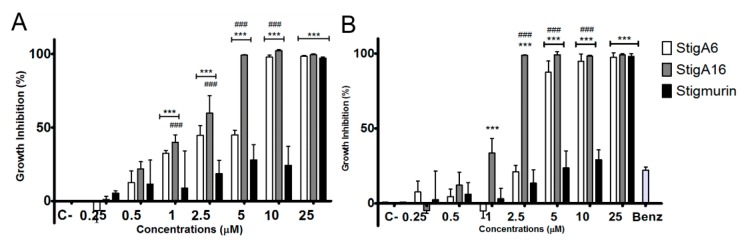
Antiparasitic activity of Stigmurin and its analogs on trypomastigotes forms of *T. cruzi* after 12 h (**A**) and 24 h (**B**) of incubation. Negative control is represented by C-. Positive control (Benznidazole) is represented by Benz. Values represent mean ± SD (N = 3). *** *p* ≤ 0.0001 compared to the positive control. ### *p* ≤ 0.0005 compared to Stigmurin at the same concentration. Statistical analysis was performed using ANOVA followed by Tukey test.

Regarding the activity against trypomastigote forms of *T. cruzi*, both analog peptides were able to inhibit approximately 100% of the parasite growth at concentrations of 10 and 25 µM after 12 h of incubation (Figure 8A). In the case of StigA16, we also observed practically 100% inhibition at 5 µM. Stigmurin was able to inhibit 100% of the parasite growth at 25 µM. After 24 h of incubation (Figure 8B), StigA6 and StigA16 were able to completely inhibit the growth at 5 µM; for StigA16, this level of inhibition was also observed at 2.5 µM. On the other hand, for Stigmurin, a high level of inhibition was obtained, after 24 h, only when parasites were incubated with 25 µM of this peptide. This implies that the analog peptides were more effective than the native peptide Stigmurin. Both analog peptides showed higher growth inhibition when compared to Benznidazole.

### 2.5. Antiproliferative Activity

**Figure 9:**
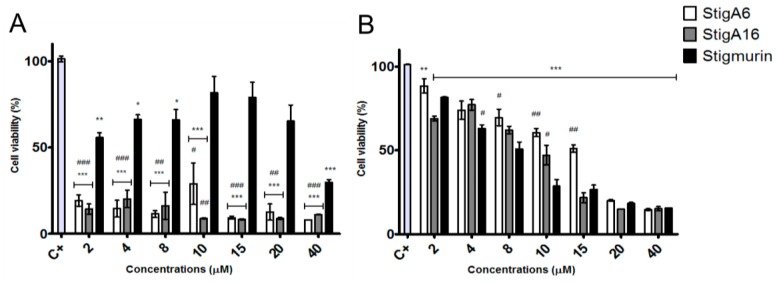
Antiproliferative activity of Stigmurin and its analogs in HeLa (**A**) and in 3T3 (**B**) cell lines. Values represent mean ± SD (N = 4). The positive control is represented as C+ (Cells without the addition of the peptides) *** *p* ≤ 0.0001, ** *p* ≤ 0.001 and * *p* ≤ 0.01 compared to the positive control. ### *p* ≤ 0.0005, ## *p* ≤ 0.005 and # *p* ≤ 0.05 compared to Stigmurin at the same concentration. Statistical analysis was performed using ANOVA followed by Tukey test.

**Figure A1:**
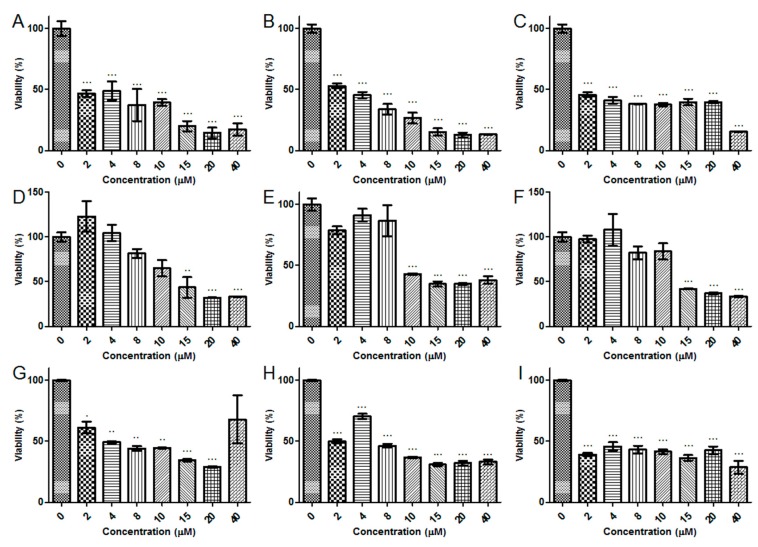
Antiproliferative activity of Stigmurin, StigA6, and StigA16 in cancerous cell lines. (**A**) StigA6 in 786-0 cell line; (**B**) StigA16 in 786-0 cell line; (**C**) Stigmurin in 786-0 cell line; (**D**) StigA6 in B16 cell line; (**E**) StigA16 in B16 cell line; (**F**) Stigmurin in B16 cell line; (**G**) StigA6 in Panc cell line; (**H**) StigA16 in Panc cell line; (**I**) Stigmurin in Panc cell line. The results are expressed as mean ± SD *** *p* < 0.0001, ** *p* ≤ 0.001 and * *p* ≤ 0.01 compared to the positive control.

StigA6 and StigA16 showed antiproliferative activity on all cancerous cell lines tested (Figure 9), presenting no significant difference among the analog peptides. In the HeLa cell line, both peptides reduced cell proliferation by 85% in all concentrations tested, while Stigmurin reduced 70% of its viability in the highest dose as shown in Figure 9A. For the normal cell line 3T3 (Figure 9B), the analog peptides exhibited the IC_50_ of 14.01 and 13.01 µM for StigA6 and StigA16, respectively, about twice the IC_50_ of Stigmurin for the same cell (IC_50_ 7.98 µM). For the other cancerous cell lines tested, no significant difference between Stigmurin and the analog peptides was observed (Appendix A Figure A1), all of them inhibiting approximately 70% the growth of 786-0, B16, and Panc cells with the highest peptide concentrations.

### 2.6. Hemolytic Activity

**Figure 10:**
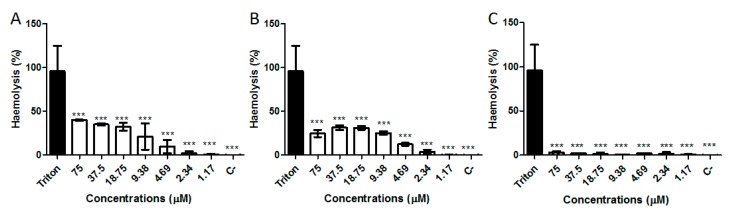
Hemolysis activity of (**A**) StigA6; (**B**) StigA16, and (**C**) Stigmurin using Triton-X as positive control. The negative control is represented as C- (Erythrocytes in PBS without Triton-X or the peptides). *** *p* < 0.0001 compared to the positive control. Statistical analysis was performed using ANOVA followed by Tukey test.

The hemolytic activity of Stigmurin, StigA6, and StigA16 in human erythrocytes was performed at concentration range of 1.17 to 75 µM (Figure 10). After one-hour incubation, Stigmurin showed low hemolytic activity (3%) at the highest dose. Analog peptides induced a percentage hemolysis of 30% when tested at the highest concentration. However, weak hemolytic activity was observed in the lower concentrations tested.

## 3. Discussion

Antimicrobial peptides have been discovered in the venom of different scorpion species, being linked to the innate immune response against pathogens [18,19,20,21,22]. In the transcriptome study of the *T. stigmurus* venom gland, an AMP denominated Stigmurin (+1 net charge and 0.571 hydrophobic moment) was identified [4,13,14]. From this peptide we designed two analog peptides, denominated StigA6 and StigA16. To design these analogs, Ser7 and Gly10 residues on the native peptide were substituted with lysine in analog StigA6, while ser3, ser7, and gly10 were substituted by lysine in StigA16; thus, we obtained peptides with higher net charge (+3 for StigA6 and +4 for StigA16) and hydrophobic moment (0.669 for StigA6 and 0.725 for StigA16), which could lead to a higher antimicrobial activity.

The secondary structure, assessed by CD, showed, for both peptides, a predominant random structure in water and PBS, but a predominant α-helix structure in SDS and in all TFE concentrations, which could also be seen for the StigA16 model after the dynamic simulation and for the StigA6 model, which showed a random coil in the water explicit simulation and an attempt for a helix structure when TFE was added to the system. The capacity to change its structure according to the environment had already been seen for other scorpion AMPs [3,13,23,24]. As these peptides usually present a predominant random coil conformation in a hydrophilic environment and a predominant α-helix structure in a hydrophobic medium, this structure flexibility can suggest their interaction with membranes, leading to pore formation and cell lysis [3,13,23,24].

Using CD, we could also observe StigA6 and StigA16 stability at pH range 3–9, as well as in temperature change; once heated to 98 °C and subsequently cooled to 2 °C, they did not appear to change their secondary structure. Stigmurin had already been seen to be stable to pH and temperature variation [13,14], indicating that the addition of lysine in the native peptide sequence did not cause impairment to the peptide stability.

Sequence changes in the peptide were efficient in improving StigA6 and StigA16 antimicrobial activity, as they could inhibit the growth of both Gram-positive (*S. aureus*, *S. epidermidis,* and *E. faecalis*) and Gram-negative (*E. cloaceae*, *P. aeruginosa,* and *E. coli*) bacteria as well as *Candida* yeasts. StigA16 showed MIC between 1.17 and 9.38 µM while StigA6 showed MIC values relatively higher, reaching 37.5 µM. In previous studies, Stigmurin had already proved its antimicrobial activity against Gram-positive bacteria and *Candida* fungi [13], but with higher MIC concentration when compared to StigA6 and StigA16, and it had not shown activity for Gram-negative bacteria, unlike the analog peptides described herein. This increase in the antimicrobial activity may be due to the analogs’ higher net charge and hydrophobic moment, which tend to increase the molecule capacity to interact with the membrane, increasing the probability of pore formation and cell lysis.

Other antimicrobial peptides from scorpions had already shown activity against Gram-positive and Gram-negative bacteria [18,25,26,27,28]. TsaP2, a peptide found in *T. serrulatus* that shows high identity with Stigmurin, presented MIC of 17.30 and 69.23 µM for *S. aureus* and *E. faecalis*, respectively; therefore, both StigA6 and StigA16 are more effective than TsaP2 [14]. The capacity of scorpion AMPs to inhibit fungal growth has also been seen [29,30]. The peptide Con10 from the scorpion *Opisthacanthus cayaporum* showed MIC of 100 and 200 µM for *C. albicans* and *C. glabrata*, respectively; therefore, it is also less efficient than the analog peptides of Stigmurin [31]. These results combined reaffirm that the StigA6 and StigA16 higher positive charge (+3 and +4) render a higher antimicrobial activity, since Stigmurin, TsaP2, and Con10 have a +1 charge. Another example is the scorpion venom analog peptide AamAP1-lysine, which showed MIC for *S. aureus*, *S. epidermidis,* and *E. faecalis* of 5 µM, while StigA6 showed MIC of 2.34 µM for *S. aureus* and 1.17 µM for *S. epidermidis* and *E. faecalis.* StigA16 showed MIC of 2.34 µM for *S. aureus* and 1.17 µM for *E. faecalis.* Additionally, for *E. coli*, AamAP1-lysine showed MIC of 7.5 µM while StigA16 showed MIC of 2.34 and StigA6, 4.69. This indicates that the analog peptides of Stigmurin show higher antimicrobial activity than AamAP1 for both Gram-positive and Gram-negative bacteria.

Regarding the antiparasitic activity, the three peptides tested showed inhibition for the *T. cruzi* Y strain using epimastigote forms, as no differences between the analog peptides were observed, although when compared with the native peptide both analog peptides exhibited higher antiparasitic activity. We also tested the peptides on the trypomastigote forms of the *T. cruzi* Y strain and we were able to observe the same pattern as that for epimastigotes, with the analog peptides showing higher activity than Stigmurin. Other venom peptides have been described as having antiparasitic activity [32,33,34]. M-PONTX-Dq3a, an AMP from the venom of the ant *Dinoponera quadriceps*, showed an IC_50_ of 4.7 µM against epimastigote *T. cruzi*, while StigA16 only needed 1 µM to inhibit approximately 50% of the parasite growth [35].

When compared to Benznidazole, at the incubation time and dose tested, both analog peptides showed higher antiparasitic activity at a lower concentration. For instance, StigA6 and Stig16 showed 100% of the trypomastigote forms of *T. cruzi* at 2.5 µM, while Benznidazole only inhibited 20% at 384 µM. It is known that Benznidazole, the main drug used in the treatment of Chagas disease, is only effective against trypomastigote forms of the *T. cruzi* Y strain after 72 h incubation [36,37]; therefore, the analog peptides were more effective at less time incubation with a minor concentration. Taking into account the peptide activity against trypomastigote forms of *T. cruzi*, which are found mainly in the blood of the patient in the acute phase of Chagas disease, we suggest that these peptides showed potential to be developed as a drug for treatment of Chagas disease.

The antiproliferative activities of Stigmurin, StigA6, and StigA16 were assessed using cancerous and normal cell lines. We could not observe a significant difference between the peptides in the cancerous lines tested; however, when the HeLa cell line was used, we observed that StigA6 and StigA16 showed higher antiproliferative activity when compared to Stigmurin. For the normal cell line tested, 3T3, both analog peptides showed an IC_50_ that was twice the value observed for Stigmurin, demonstrating that they are less toxic for this normal cell than the native peptide. Other scorpion AMPs demonstrated activity on cancerous and normal cells [14,15,38]. A study with analog scorpion AMPs showed that the peptides with lysine substitutions were less effective on the BHK21 normal cell [38]. The result of this study, combined with what was observed for StigA6 and StigA16, may indicate that the lysine addition makes this class of peptides more selective and, consequently, less toxic to normal cells.

The hemolytic activity revealed that Stigmurin did not show significant hemolysis in all tested doses when incubated for one hour, and, despite the significant difference between Stigmurin and its analogs, StigA6 and StigA16 showed approximately 30% hemolysis at 75 µM. This increase in hemolysis activity can be explained by the addition of lysine in the peptide sequence, which increases the peptide hydrophobic moment and, thus, its interaction with the erythrocyte membrane [15,17]. It should be highlighted, however, that at lower concentrations, in which the analog peptides showed antimicrobial and antiproliferative activities, they showed minimum hemolysis (lower than 10%).

## 4. Conclusions

In this study, we report the modifications in the Stigmurin sequence to generate StigA6 and StigA16, which were efficient at enhancing the antimicrobial activity against Gram-positive and yeasts, also increasing the spectrum on Gram-negative bacteria. The lysine substitutions in StigA6 and StigA16, which led to higher net charge and hydrophobic moment, did not affect the stability at temperature and pH conditions, when compared to the native peptide. The peptides also showed higher antiparasitic activity against epimastigote and trypomastigote *T. cruzi*. The analog peptides showed activity against cancerous cells, but they were less toxic to the normal cells tested than Stigmurin. Therefore, these analog peptides are molecules with high biotechnological potential, proving that rational design is a promising tool to obtain molecules for therapeutic application.

## 5. Materials and Methods

### 5.1. Peptide Synthesis

**Figure A2:**
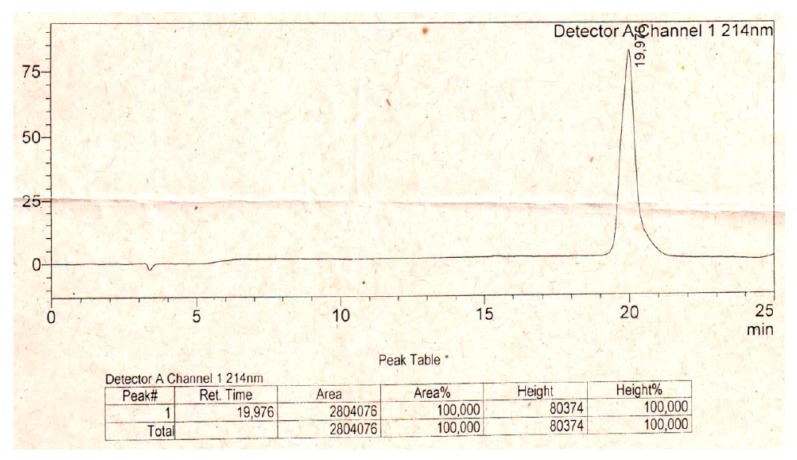
High performance liquid chromatography (HPLC) spectrum of StigA6.

**Figure A3:**
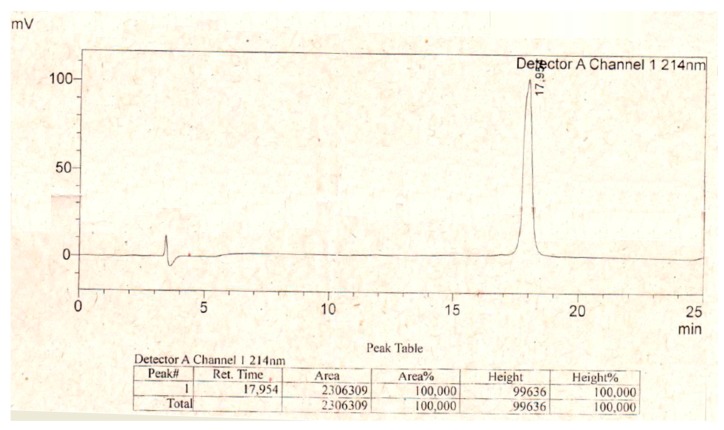
High performance liquid chromatography (HPLC) spectrum of StigA16.

**Figure A4:**
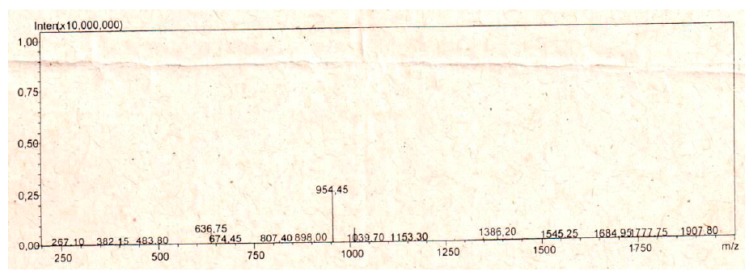
Mass spectrometry spectrum of StigA6.

**Figure A5:**
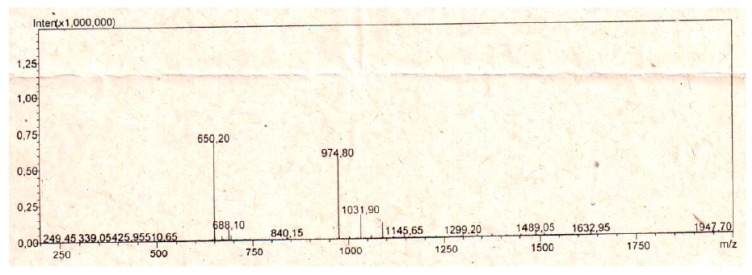
Mass spectrometry spectrum of StigA16.

C-terminal amidated StigA6 (1907.42 Da) and StigA16 (1948.51 Da) were commercially synthesized by Aminotech (Minas Gerais, Brazil) and stored at −80 °C until use. Peptide masses were assessed by electrospray ionization mass spectrometry and their purity confirmed by high performance liquid chromatography (HPLC) (>90% purity). HPLC (Figure A2 and Figure A3) and mass spectrometry (Figure A4 and Figure A5) plots are shown in Appendix for StigA6 and StigA16, respectively.

### 5.2. In Silico Structural Analysis

The evaluation of physicochemical parameters (net charge and hydrophobic moment) of both peptides was assessed with the Heliquest server (http://heliquest.ipmc.cnrs.fr/). StigA6 and StigA16 modelling was performed using the I-TASSER (https://zhanglab.ccmb.med.umich.edu/I-TASSER/) server and structures were validated by Ramachandran plots, β carbon derivation, bonds and angles using MolProbity [39]. PDB files were visualized using USFC Chimera software (Version 1.8.1, San Francisco, CA, USA, 2013) [40].

For the molecular dynamics simulations, the amidated model topologies were defined by CHARMM27 force field [41]. The simulations were performed using GROMACS 5.1.4 software (San Francisco, CA, USA, 2016) [42] with the water explicit model TIP3P and with 50%TFE solution. The models were submitted to energy and temperature minimization and the simulations were held with 298 K and 1 bar for 0.5 microseconds.

### 5.3. Analysis of Secondary Structure and Stability by Circular Dichroism

StigA6 and StigA16 peptides were evaluated by circular dichroism (CD) on a spectropolarimeter JASCO-810 at 25 °C using a Peltier system. The scan range of wavelengths was from 182 nm to 260 nm at 50 nm·min^−1^. The CD spectra were measured by averaging five scans. Both analogs were analyzed in water, sodium phosphate buffer (PBS), 20 mM sodium dodecyl sulfate (SDS) or in 2,2,2-trifluoroethanol (TFE) at 30, 40, 50, 60, and 70% (*v*/*v*). The spectra were presented in molar ellipticity and the secondary structure percentage was obtained by deconvolution of the spectrum with Dichroweb server (http://dichroweb.cryst.bbk.ac.uk/html/home.shtml) using Selcon3 and CONTIN II algorithms [43,44]. To evaluate the stability of StigA6 and StigA16, they were submitted to pH analysis (ranging from 3–9) at 20 mM SDS and 40% TFE. The influence of temperature was assessed by two assays, in the first one, the peptides were analyzed from 182 nm to 260 nm at 5, 25, 37, 50, 75, and 95 °C, in the second one, the peptides were analyzed at 222 nm and were heated from 2 to 98 °C and then cooled back to 2 °C.

### 5.4. Antimicrobial Activity

For antimicrobial assays, a panel of microorganisms were used, including Gram-positive bacteria: *Staphylococcus aureus* (ATCC 29213), *Staphylococcus epidermidis* (ATCC 12228), and *Enterococcus faecalis* (ATCC 4028); Gram-negative bacteria: *Escherichia coli* (ATCC 25922), *Enterobacter cloacae* (ATCC 13047), and *Pseudomonas aeruginosa* (ATCC 27853); and yeasts: *Candida albicans* (ATCC 90028), *Candida krusei* (ATCC 6258), and *Candida glabrata* (ATCC 90030). Minimal inhibitory concentration (MIC) was determined by broth microdilution method in Muller-Hinton broth (MHB), as described in the Clinical and Laboratory Standards Institute (CLSI). The inoculum was prepared at 1 × 10^5^ colony forming unit per milliliter (CFU/mL) for bacteria and 1 × 10^4^ CFU/mL for yeasts. Cells were grown overnight at 35 °C and, afterwards, 50 µL of the microbe suspension was added to serial dilutions of the peptides (final concentrations 1.17–75 µM, assay total volume corresponded to 100 µL). The assay was done in 96-well microplate and the samples were incubated at 35 °C for 24 h. The microbial growth was assessed by measurements of the A595 nm in a microplate reader (Epoch, BioTek Instruments, Winooski, VT, USA). Vancomycin, Gentamicin, and Amphotericin B were used as positive control for Gram-positive bacteria, Gram-negative bacteria, and Yeasts, respectively, following CLSI guidelines. The negative control was processed under identical conditions without addition of the peptides nor the standard antibiotics and used as control for 100% bacterial growth. MIC was defined as the lowest concentration able to prevent microbial growth. The assays were made in triplicate.

### 5.5. Antiparasitic Activity

The antiparasitic activity against the *Trypanosoma cruzi* Y strain was performed using epimastigote and trypomastigote forms. For the epimastigote form, the parasites were incubated for 11 days at 27 ± 2 °C in Liver Infusion Tryptose (LIT) medium until stationary phase, and then incubated with the antimicrobial peptides at different concentrations (0.25–25 µM) for 12 and 24 h at 27 ± 2 °C in 96-well plates. Subsequently, 3-(4,5-dimethylthiazol-2-yl)-2,5-diphenyltetrazolium bromide (MTT) was added and incubated for 75 min and, after this period, HCL 0.01 N with 10% SDS was added and incubated for 30 min to solubilize the formazan crystals, and then the plate was read at 595 nm. Benznidazole (384 µM) was used as positive control at 12 and 24 h of incubation. The negative control, without addition of the peptides, was used as control for 100% parasite viability.

For the trypomastigote assays, initially the epimastigote parasites were transformed into trypomastigotes using a 1:9 dilution of the epimastigote solution, and then incubated for 25 days at 27 ± 2 °C. The tests were performed following the methodology described for the epimastigote form.

### 5.6. Antiproliferative Activity

The cytotoxicities of the synthetic peptides were evaluated in human renal cell adenocarcinoma (786-0, ATCC^®^ CRL-1932), mouse melanoma (B16­F10, ATCC^®^ CRL­6475), human cervix adenocarcinoma (HeLa, ATCC^®^ CCL-2), human pancreas adenocarcinoma (Panc 10.05, ATCC CRL-2547), and the normal cell line from mouse fibroblast (NIH/3T3, ATCC^®^ CRL-1658). Cell viability was measured by 3-(4,5-dimethylthiazol-2-yl)-2,5-diphenyltetra-zoliumbromide (MTT) assay. Confluent cell-monolayers contained in 96-well plates were incubated with serially diluted Stigmurin, StigA6, or StigA16 (2–40 µM) in Dulbecco’s Modified Eagle Medium (DMEM) medium for B16-F10, HeLa, Panc, and 3T3, or Roswell Park Memorial Institute Medium (RPMI) medium for 786-0 cells. Plates were incubated at 37 °C for 24 h. The MTT solution (1 mg/mL) was added to each well and further incubated for 4 h at 37 °C. Supernatants were removed and replaced by 96% ethanol (*v*/*v*) in order to solubilize the formazan crystals. The absorbance of the plate was measured at 570 nm. The positive control, without addition of the peptides, was used as control for 100% parasite viability.

### 5.7. Hemolytic Activity

Hemolytic activity of StigA6, StigA16, and Stigmurin was carried out by incubating a suspension of healthy human donor O^+^ erythrocytes with serially diluted concentrations of the peptides. Cells were first washed three times by centrifugation at 2000 RPM for 10 min in PBS, then incubated with the synthetic peptides (1.17–75 µM) at 37 °C for 1 h [45]. Optical density of supernatants was measured at 540 nm using a microplate reader. Triton was used as positive control, and no compost was added to the negative control for 0% hemolysis.

### 5.8. Statistical Analysis

All experimental values were expressed as mean ± standard deviation (SD). One-way analysis of variance (ANOVA) was applied for multiple-group comparisons, followed by the post-test of Tukey using GraphPad Prism software (Version 5.00, GraphPad, San Diego, CA, USA, 2007). A value of *p* < 0.05 was considered to be statistically significant.

## Figures and Tables

**Table 1:** Secondary structure analysis of StigA6 and StigA16 in water, PBS, SDS, and TFE (20–70%).

	StigA6			StigA16		
	α-Helix (%)	β-Sheet (%)	Random (%)	α-Helix (%)	β-Sheet (%)	Random (%)
Water	5.65 ± 4.8	14.1 ± 4.5	78 ± 12.5	1.45 ± 0.7	13.75 ± 1.06	82.45 ± 1.4
PBS	4.55 ± 0.7	9.6 ± 0.5	86.05 ± 0.4	8.6 ± 8	15.2 ± 0.9	76.9 ± 10
SDS 20 mM	66.6 ± 2.1	2.15 ± 0.2	32.05 ± 2.8	58.8 ± 0.8	5.25 ± 0.2	35.55 ± 1.7
TFE 20%	47.7 ± 1.5	9.4 ± 3.1	42.6 ± 2.1	49.9 ± 2.5	10.45 ± 4.5	45.1 ± 1.2
TFE 30%	56.7 ± 3.3	7.15 ± 0.3	36.1 ± 3.1	50.75 ± 1.2	9.6 ± 3.2	39.85 ± 1.6
TFE 40%	58.4 ± 2.8	5.55 ± 0.6	35.5 ± 2.9	55.85 ± 0.4	5.55 ± 0.9	39.05 ± 0.2
TFE 50%	65.8 ± 13.4	4.2 ± 4.9	30.3 ± 8.9	52.5 ± 1.4	7.55 ± 1.3	40.1 ± 0.1
TFE 60%	52.95 ± 4.3	7.55 ± 2.4	39.8 ± 2.1	49.6 ± 0.6	5.55 0.2	46 ± 2.2
TFE 70%	54.3 ± 3.1	7.85 ± 2	38.15 ± 1.6	49.75	4.8 ± 0.7	45.7 ± 0.8

**Table 2:** Minimal inhibitory concentration (MIC, in µM) of StigA6 and StigA16 for Gram-positive and -negative bacteria and yeasts.

Strains	StigA6 (µM)	StigA16 (µM)	Stigmurin (µM)
Gram-negative bacteria			
*Escherichia coli* (ATCC 25922)	4.69	2.34	>150
*Enterobacter cloacae* (ATCC 13047)	18.75	9.38	>150
*Pseudomonas aeruginosa* (ATCC 27853)	9.38	1.17	>150
Gram-positive bacteria			
*Staphylococcus aureus* (ATCC 29213)	2.34	2.34	9.38
*Staphylococcus epidermidis* (ATCC 122225)	1.17	9.38	9.38
*Enterococcus faecalis* (ATCC 4028)	1.17	1.17	>150
Yeasts			
*Candida albicans* (ATCC 90028)	9.38	4.69	37.5
*Candida krusei* (ATCC 6258)	37.5	9.38	>150
*Candida glabrata* (ATCC 90030)	18.75	9.38	>150

## References

[1] Jeram A.J., Selden P.A. (1997). Phylogeny, classification and evolution of Silurian and Devonian scorpions. Proceedings of the 17th European Colloquium of Arachnology, Edinburgh, UK, 14–18 July 1997.

[2] Waddington J., Rudkin D.M., Dunlop J.A. (2015). A new mid-Silurian aquatic scorpion—One step closer to land?. Biol. Lett..

[3] Almaaytah A., Albalas Q. (2014). Scorpion venom peptides with no disulfide bridges: A review. Peptides.

[4] Almeida D.D., Scortecci K.C., Kobashi L.S., Agnez-Lima L.F., Medeiros S.R.B., Silva-Junior A.A., Junqueira-de-Azevedo Ide L., Fernandes-Pedrosa Mde F. (2012). Profiling the resting venom gland of the scorpion *Tityus stigmurus* through a transcriptomic survey. BMC Genom..

[5] Du Q., Hou X., Wang L., Zhang Y., Xi X., Wang H., Zhou M., Duan J., Wei M., Chen T. (2015). AaeAP1 and AaeAP2: Novel antimicrobial peptides from the venom of the scorpion, *Androctonus aeneas*: Structural characterisation, molecular cloning of biosynthetic precursor-encoding cDNAs and engineering of analogues with enhanced antimicrobial and anticancer activities. Toxins.

[6] Lakshmaiah Narayana J., Chen J.-Y. (2015). Antimicrobial peptides: Possible anti-infective agents. Peptides.

[7] Sierra J.M., Fusté E., Rabanal F., Vinuesa T., Viñas M. (2017). An overview of antimicrobial peptides and the latest advances in their development. Expert Opin. Biol. Ther..

[8] Toke O. (2005). Antimicrobial peptides: New candidates in the fight against bacterial infections. Biopolymers.

[9] Uematsu N., Matsuzaki K. (2000). Polar angle as a determinant of amphipathic alpha-helix-lipid interactions: A model peptide study. Biophys. J..

[10] Velasco-Bolom J.-L., Corzo G., Garduño-Juárez R. (2017). Molecular dynamics simulation of the membrane binding and disruption mechanisms by antimicrobial scorpion venom-derived peptides. J. Biomol. Struct. Dyn..

[11] Yeaman M.R., Yount N.Y. (2003). Mechanisms of antimicrobial peptide action and resistance. Pharmacol. Rev..

[12] McGann P., Snesrud E., Maybank R., Corey B., Ong A.C., Clifford R., Hinkle M., Whitman T., Lesho E., Schaecher K.E. (2016). *Escherichia coli* Harboring mcr-1 and blaCTX-M on a Novel IncF Plasmid: First Report of mcr-1 in the United States. Antimicrob. Agents Chemother..

[13] Melo E.T., Estrela A.B., Santos E.C.G., Machado P.R.L., Farias K.J.S., Torres T.M., Carvalho E., Lima J.P.M.S., Silva-Júnior A.A., Barbosa E.G. (2015). Structural characterization of a novel peptide with antimicrobial activity from the venom gland of the scorpion *Tityus stigmurus*: Stigmurin. Peptides.

[14] Daniele-Silva A., Machado R.J.A., Monteiro N.K.V., Estrela A.B., Santos E.C.G., Carvalho E., Araújo Júnior R.F., Melo-Silveira R.F., Rocha H.A.O., Silva-Júnior A.A. (2016). Stigmurin and TsAP-2 from *Tityus stigmurus* scorpion venom: Assessment of structure and therapeutic potential in experimental sepsis. Toxicon.

[15] Almaaytah A., Tarazi S., Abu-Alhaijaa A., Altall Y., Alshar’i N., Bodoor K., Al-Balas Q. (2014). Enhanced Antimicrobial Activity of AamAP1-Lysine, a Novel Synthetic Peptide Analog Derived from the Scorpion Venom Peptide AamAP1. Pharmaceuticals.

[16] Salud Bea R., Ascuitto M.R., de Johnson L.E.L. (2015). Synthesis of analogs of peptides from *Buthus martensii* scorpion venom with potential antibiotic activity. Peptides.

[17] Salud Bea R., Petraglia A.F., Ascuitto M.R., Buck Q.M. (2017). Antibacterial Activity and Toxicity of Analogs of Scorpion Venom IsCT Peptides. Antibiotics.

[18] Almaaytah A., Zhou M., Wang L., Chen T., Walker B., Shaw C. (2012). Antimicrobial/cytolytic peptides from the venom of the North African scorpion, *Androctonus amoreuxi*: Biochemical and functional characterization of natural peptides and a single site-substituted analog. Peptides.

[19] Guo X., Ma C., Du Q., Wei R., Wang L., Zhou M., Chen T., Shaw C. (2013). Two peptides, TsAP-1 and TsAP-2, from the venom of the Brazilian yellow scorpion, *Tityus serrulatus*: Evaluation of their antimicrobial and anticancer activities. Biochimie.

[20] Mandard N., Sy D., Maufrais C., Bonmatin J.M., Bulet P., Hetru C., Vovelle F. (1999). Androctonin, a novel antimicrobial peptide from scorpion *Androctonus australis*: Solution structure and molecular dynamics simulations in the presence of a lipid monolayer. J. Biomol. Struct. Dyn..

[21] Torres-Larios A., Gurrola G.B., Zamudio F.Z., Possani L.D. (2000). Hadrurin, a new antimicrobial peptide from the venom of the scorpion *Hadrurus aztecus*. Eur. J. Biochem..

[22] Zhao Z., Ma Y., Dai C., Zhao R., Li S., Wu Y., Cao Z., Li W. (2009). Imcroporin, a new cationic antimicrobial peptide from the venom of the scorpion *Isometrus maculates*. Antimicrob. Agents Chemother..

[23] Zasloff M. (2002). Antimicrobial peptides of multicellular organisms. Nature.

[24] Leite N.B., Aufderhorst-Roberts A., Palma M.S., Connell S.D., Ruggiero Neto J., Beales P.A. (2015). PE and PS Lipids Synergistically Enhance Membrane Poration by a Peptide with Anticancer Properties. Biophys. J..

[25] Dai L., Yasuda A., Naoki H., Corzo G., Andriantsiferana M., Nakajima T. (2001). IsCT, a novel cytotoxic linear peptide from scorpion *Opisthacanthus madagascariensis*. Biochem. Biophys. Res. Commun..

[26] Lee K., Shin S.Y., Kim K., Lim S.S., Hahm K.-S., Kim Y. (2004). Antibiotic activity and structural analysis of the scorpion-derived antimicrobial peptide IsCT and its analogs. Biochem. Biophys. Res. Commun..

[27] Li Z., Xu X., Meng L., Zhang Q., Cao L., Li W., Wu Y., Cao Z. (2014). Hp1404, a new antimicrobial peptide from the scorpion *Heterometrus petersii*. PLoS ONE.

[28] Cao L., Dai C., Li Z., Fan Z., Song Y., Wu Y., Cao Z., Li W. (2012). Antibacterial activity and mechanism of a scorpion venom peptide derivative in vitro and in vivo. PLoS ONE.

[29] Ehret-Sabatier L., Loew D., Goyffon M., Fehlbaum P., Hoffmann J.A., van Dorsselaer A., Bulet P. (1996). Characterization of novel cysteine-rich antimicrobial peptides from scorpion blood. J. Biol. Chem..

[30] Moerman L., Bosteels S., Noppe W., Willems J., Clynen E., Schoofs L., Thevissen K., Tytgat J., Van Eldere J., Van Der Walt J. (2002). Antibacterial and antifungal properties of alpha-helical, cationic peptides in the venom of scorpions from southern Africa. Eur. J. Biochem..

[31] Guilhelmelli F., Vilela N., Smidt K.S., de Oliveira M.A., da Cunha Morales Álvares A., Rigonatto M.C.L., da Silva Costa P.H., Tavares A.H., de Freitas S.M., Nicola A.M. (2016). Activity of Scorpion Venom-Derived Antifungal Peptides against Planktonic Cells of Candida spp. and Cryptococcus neoformans and Candida albicans Biofilms. Front. Microbiol..

[32] Borges A., Silva S., Op den Camp H.J.M., Velasco E., Alvarez M., Alfonzo M.J.M., Jorquera A., De Sousa L., Delgado O. (2006). In vitro leishmanicidal activity of *Tityus discrepans* scorpion venom. Parasitol. Res..

[33] Flores-Solis D., Toledano Y., Rodríguez-Lima O., Cano-Sánchez P., Ramírez-Cordero B.E., Landa A., Rodríguez de la Vega R.C., Del Rio-Portilla F. (2016). Solution structure and antiparasitic activity of scorpine-like peptides from Hoffmannihadrurus gertschi. FEBS Lett..

[34] Conde R., Zamudio F.Z., Rodríguez M.H., Possani L.D. (2000). Scorpine, an anti-malaria and anti-bacterial agent purified from scorpion venom. FEBS Lett..

[35] Bandeira L.D., Perdigão M.C., Justino B.I.C., Pessoa Bezerra de Menezes R.R.P., Lima S.T., Borges F.C., Morlighem J.-É.R.L., Rádis-Baptista G., Costa M.A.M. (2017). The dinoponeratoxin peptides from the giant ant *Dinoponera quadriceps* display in vitro antitrypanosomal activity. Biol. Chem..

[36] Muelas-Serrano S., Nogal-Ruiz J.J., Gómez-Barrio A. (2000). Setting of a colorimetric method to determine the viability of *Trypanosoma cruzi* epimastigotes. Parasitol. Res..

[37] Zingales B. (2017). *Trypanosoma cruzi* genetic diversity: Something new for something known about Chagas disease manifestations, serodiagnosis and drug sensitivity. Acta Trop..

[38] Luna-Ramirez K., Tonk M., Rahnamaeian M., Vilcinskas A. (2017). Bioactivity of Natural and Engineered Antimicrobial Peptides from Venom of the Scorpions *Urodacus yaschenkoi* and *U. manicatus*. Toxins.

[39] Chen V.B., Arendall W.B., Headd J.J., Keedy D.A., Immormino R.M., Kapral G.J., Murray L.W., Richardson J.S., Richardson D.C. (2010). MolProbity: All-atom structure validation for macromolecular crystallography. Acta Crystallogr. D Biol. Crystallogr..

[40] Pettersen E.F., Goddard T.D., Huang C.C., Couch G.S., Greenblatt D.M., Meng E.C., Ferrin T.E. (2004). UCSF Chimera—A visualization system for exploratory research and analysis. J. Comput. Chem..

[41] Vanommeslaeghe K., Hatcher E., Acharya C., Kundu S., Zhong S., Shim J., Darian E., Guvench O., Lopes P., Vorobyov I. (2010). CHARMM general force field: A force field for drug-like molecules compatible with the CHARMM all-atom additive biological force fields. J. Comput. Chem..

[42] Van Der Spoel D., Lindahl E., Hess B., Groenhof G., Mark A.E., Berendsen H.J.C. (2005). GROMACS: Fast, flexible, and free. J. Comput. Chem..

[43] Sreerama N., Venyaminov S.Y., Woody R.W. (1999). Estimation of the number of alpha-helical and beta-strand segments in proteins using circular dichroism spectroscopy. Protein Sci. Publ. Protein Soc..

[44] Van Stokkum I.H., Spoelder H.J., Bloemendal M., van Grondelle R., Groen F.C. (1990). Estimation of protein secondary structure and error analysis from circular dichroism spectra. Anal. Biochem..

[45] Menezes Y.A.S., Félix-Silva J., da Silva-Júnior A.A., Rebecchi I.M.M., de Oliveira A.S., Uchoa A.F., Fernandes-Pedrosa Mde F. (2014). Protein-rich fraction of Cnidoscolus urens (L.) Arthur leaves: Enzymatic characterization and procoagulant and fibrinogenolytic activities. Molecules.

